# A Further Analysis of the Relationship between Yellow Ripe-Fruit Color and the Capsanthin-Capsorubin Synthase Gene in Pepper (*Capsicum* sp.) Indicated a New Mutant Variant in *C. annuum* and a Tandem Repeat Structure in Promoter Region

**DOI:** 10.1371/journal.pone.0061996

**Published:** 2013-04-18

**Authors:** Zheng Li, Shu Wang, Xiao-Ling Gui, Xiao-Bei Chang, Zhen-Hui Gong

**Affiliations:** 1 College of Horticulture, Northwest A&F University, Yangling, Shaanxi, China; 2 College of Horticulture and Landscape Architecture, Southwest Forest University, Kunming, Yunnan, China; Centro de Investigación y de Estudios Avanzados del IPN, Mexico

## Abstract

Mature pepper (*Capsicum* sp.) fruits come in a variety of colors, including red, orange, yellow, brown, and white. To better understand the genetic and regulatory relationships between the yellow fruit phenotype and the capsanthin-capsorubin synthase gene (*Ccs*), we examined 156 *Capsicum* varieties, most of which were collected from Northwest Chinese landraces. A new *ccs* variant was identified in the yellow fruit cultivar CK7. Cluster analysis revealed that CK7, which belongs to the *C. annuum* species, has low genetic similarity to other yellow *C. annuum* varieties. In the coding sequence of this *ccs* allele, we detected a premature stop codon derived from a C to G change, as well as a downstream frame-shift caused by a 1-bp nucleotide deletion. In addition, the expression of the gene was detected in mature CK7 fruit. Furthermore, the promoter sequences of *Ccs* from some pepper varieties were examined, and we detected a 176-bp tandem repeat sequence in the promoter region. In all *C. annuum* varieties examined in this study, the repeat number was three, compared with four in two *C. chinense* accessions. The sequence similarity ranged from 84.8% to 97.7% among the four types of repeats, and some putative *cis*-elements were also found in every repeat. This suggests that the transcriptional regulation of *Ccs* expression is complex. Based on the analysis of the novel *C. annuum* mutation reported here, along with the studies of three mutation types in yellow *C. annuum* and *C. chinense* accessions, we suggest that the mechanism leading to the production of yellow color fruit may be not as complex as that leading to orange fruit production.

## Introduction

Plants have colored flowers and fruits to attract insects or other animals acting as pollinators and seed dispersers [1]. The *Capsicum* genus, which is generally accepted to originate in South America, comprises 25–30 species [2]. Five of these species have been domesticated, including *C. annuum*, *C. baccatum*, *C. chinense*, *C. frutescens* and *C. pubescens*. The species with the greatest number of cultivated varieties worldwide is *C. annuum*. The wild progenitor of *C. annuum* is thought to be the bird pepper, whose fruit is small and red when ripe [2]. The ripe color of pepper has undergone selection during domestication, which has resulted in some new phenotypes, including yellow, orange, brown and even white fruits, which are found in all cultivated pepper species [3]. Since diverse colors (from white to red) can found across *Capsicum* varieties, pepper has become a good model system for studying the mechanism of fruit color change [3].

The color found in ripe pepper fruit (*Capsicum* sp.) mainly depends on the variable accumulation of capsanthin and capsorubin, both of which are involved in the carotenoid biosynthetic pathway. Red peppers accumulate higher levels of total carotenoid during ripening [4]. Some studies have indicated that the expression levels of carotenoid biosynthesis-related genes are directly linked to high total carotenoid accumulation in *Capsicum*. The expression levels of some carotenoid biosynthetic gene, including phytoene synthase (*Psy*), phytoene desaturase (*Pds*) and capsanthin-capsorubin synthase (*Ccs*), are relatively high in red peppers, whereas some of these genes are not expressed in peppers with lower levels of total carotenoid [4].

Test crosses between red and white-fruited varieties indicated that the inheritance of mature fruit color is controlled by three loci—*c1*, *c2* and *y* [5], [6]. The presence of dominant alleles at all three loci leads to the production of red ripe-color fruit [7]. The *c2* locus is firstly considered as a major locus for orange fruit color and is associated with the *Psy* gene [8], [9]. The deletion of the upstream region of the *Ccs* gene causes a second orange color phenotype [10]. Additionally, in the orange *C. annuum* cultivar Fogo, a mutation in *Ccs* produces premature termination [11]. Recently, Rodriguez-Uribe *et al.* [12] suggested that the control of color development in orange *C. annuum* involves a more complex process than the presence of a deletion in a structural gene for a step in pigment biosyntheses; orange ripe color may involve transcriptional regulation of *Psy* and/or *Ccs* expression.

The yellow pepper fruit color phenotype, which is recessive to red, is controlled by the *y* locus. Linkage analysis indicated that the candidate gene of the *y* locus is also the *Ccs* gene [4], [13]. To the best of our knowledge, three types of mutations for the *Ccs* gene have been found in yellow fruit pepper, including the following: a deletion that lacks the distal 220 bp of the 3′ end of the gene in *C. annuum* cultivars [7], [13], a premature stop-codon and a frame-shift in the coding sequence in two *C. chinense* accessions [4].

Since diverse modes of regulation have been found to occur between *Ccs* and the orange color phenotype, further studies about the relationship between *Ccs* and yellow color ripe-fruit are needed. Here, we identified a new mutant *ccs* variant in the yellow fruit cultivar CK7, which was derived from a local Northwest Chinese pepper population. Cluster analysis was used to examine the genetic relationship between members of the population. We found that CK7, which is a *C. annuum* species, shares low genetic similarity with other yellow *C. annuum* varieties. In the coding sequence of the *ccs* allele of CK7, we detected a premature stop codon derived from a C to G change, along with a downstream frame-shift caused by a 1-bp deletion. The second mutant position was the same as the type found in the Fogo cultivar [11]. In addition, we detected the expression of *ccs* in CK7, which was also detected in orange Fogo fruits [12]. Since the differential expression of *Ccs* can influence fruit color, we analyzed the promoter sequences of this gene in various pepper varieties. Unexpectedly, a 176-bp tandem repeat sequence was found in the promoter regions. In all *C. annuum* varieties studied, the repeat number was three, compared with four in two *C. chinense* accessions, thus leading to the presence of a relatively long promoter sequence in the *C. chinense* accessions. The level of similarity among the four repeat types ranged from 84.8% to 97.7%, and some putative *cis*-elements were also found in all of the repeats. These results suggest that there is complex transcriptional regulation of *Ccs* expression. The results of the novel mutation found in *C. annuum* and three other types of mutations in *C. annuum* and *C. chinense* accessions suggest that yellow fruit color is controlled by one structural gene in *Capsicum*.

## Materials and Methods

### Plant materials

All the 156 pepper cultivars were obtained from the College of Horticulture, Northwest Agriculture & Forestry University. Among these cultivars, 14 accessions were selected from the Asian Vegetable Research and Development Center (Table 1). All cultivars were maintained by inbreeding at the Horticulture Farm in Yangling, China. Twelve plants per line were grown in greenhouses from March to July, under natural light, at a maximum and minimum temperature of 35 and 16°C, respectively. The fruits were harvested both at the immature (nearly 30 days after anthesis and before the breaking period) and fully mature (more than 40 days after anthesis, with the final fruit color fixed) stages. For each stage, more than five fruits were collected from the same pepper lines and frozen in liquid nitrogen for subsequent RNA extractions. The fruit colors were observed and photographed at the mature stage. Young leaves were used for DNA extractions.

**Table 1:** Descriptive characteristics of pepper cultivars, developed by the Asian Vegetable Research and Development Center (AVRDC-The World Vegetable Center).

Name in this study	Vegetable introduction number in AVRDC	Genus and species	Country of collection	Color at mature stage
R11	VI059346	*C. annuum*	USA	Red
R12	VI012493	*C. annuum*	Canada	Yellow
R13	VI012712	*C. annuum*	Bulgaria	Red
R14	VI012755	*C. sp*	Korea	Red
R15	VI047076	*C. annuum*	Canada	Red
R19	VI012852	*C. annuum*	USA	Yellow
R25	VI047102	*C. annuum*	USA	Red
R26	VI046805	*C. baccatum*	Peru	Red
R28	VI044315	*C. chinense*	Unknown	Red
R29	VI046935	*C. frutescens*	Mexico	Red
R30	VI047018	*C. chinense*	USA	Red
R34	VI027861	*C. annuum*	Turkey	Red
R37	VI012716	*C. annuum*	Bulgaria	Red
R38	VI012496	*C. baccatum*	Chile	Red

### SSR analysis

DNA from young leaves was extracted as described by Li *et al*. [14]. A total of 25 genomic SSR loci [15] and seven other SSR markers (suggested by Prof. Alain Palloix, INRA, France) were used to analyze the phylogenetic relationship between the cultivars (Table S1). PCR was performed as follows: 35 cycles at 94°C for 30 s, 55°C for 30 s, and 72°C for 30 s. The PCR products were analyzed in 6% denatured polyacrylamide gels in 1 × TBE [16].

### Data analysis

Each fingerprint profile was scored for the presence or absence of bands in a gel. Pairwise genetic similarities among individuals were computed with NTSYSpc 2.02j [17]. Pairwise genetic distances were averaged among individuals within each accession to generate an accession pairwise genetic distance matrix. Dendrograms were constructed using the UPGMA method (Unweighted Pair Group Method with Arithmetic mean).

### Cloning of *Ccs* gene and promoter sequences

Sixteen genetically distinct pepper cultivars were selected based on the color types in fully ripe fruit (eight red and eight yellow), including CK6 (*C. annuum*, red), CK8 (*C. annuum*, red), R29 (*C. frutescens*, red), R37 (*C. annuum*, red), R28 (*C. chinense*, red), R30 (*C. chinense*, red), R26 (*C. baccatum*, red), R38 (*C. baccatum*, red), P123-1-1 (*C. annuum*, yellow), CK4 (*C. annuum*, yellow), CK4-1 (*C. annuum*, yellow), CK18 (*C. annuum*, yellow), CK7 (*C. annuum*, yellow), CK26-1 (*C. annuum*, yellow), R12 (*C. annuum*, yellow), and R19 (*C. annuum*, yellow). The CDS (coding sequence) and promoter fragments of the *Ccs* gene from all sixteen cultivars were amplified using a Long-Distance PCR Kit (Takara, China) with gene-specific primers (Table S2). The resulting PCR products were subcloned and sequenced as described previously [14].

Multiple sequence alignment of full-length CDS was performed using ClustalX software (http://www.ch.embnet.org/software/clustal_X.html) and displayed using the DNAMAN 4.0 program (Lynnon Biosoft. Co.). Phylogenetic trees were constructed using MEGA3.1 software (http://www.megasoftware.net/) based on the neighbor-joining method. Bootstrap values were calculated from 1,000 trials.

The promoter fragments from pepper lines CK8 (*C. annuum*), R28 (*C. chinense*) and R30 (*C. chinense*) were compared to identify the homologous tandem repeat regions and *cis*-acting elements. Putative *cis*-acting elements were identified by searching the PLACE database (http://www.dna.affrc.go.jp/PLACE/) [18] and PlantCARE database (http://bioinformatics.psb.ugent.be/webtools/plantcare/html/).

### Expression of *Ccs*

The expression of *Ccs* gene was analyzed using quantitative RT-PCR. Total RNA was extracted from immature and ripe red/yellow pericarps using a previously described method [16], [19]. PCR was performed in a 96-well plate using an ABI 7500 Fast Real-Time PCR System (Applied Biosystems, USA), with SYBR Green Realtime PCR Master Mix (TaKaRa, China). The amplification was initiated by heating to 94°C for 10 min followed by 40 cycles at 94°C for 5 s and 62°C for 30 s. The amplification specificity was tested by producing a dissociation curve (65°C to 90°C). To compare results from different reactions and samples, *Ccs* amplification was normalized to that of the *ubiquitin* gene using the CT values [20]. The expression results represent the average of three independent biological replicates from different fruits of a single plant.

## Results

### Color phenotypes and cluster analysis of pepper cultivars

**Figure 1:**
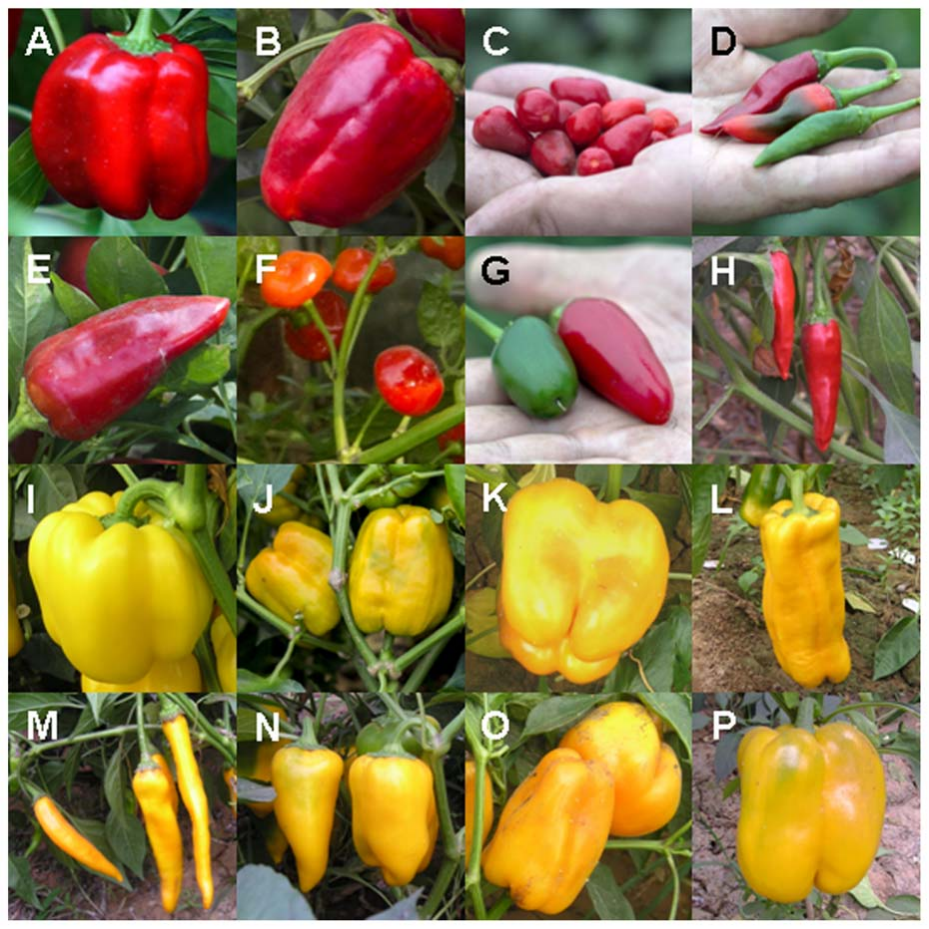
Mature red and yellow pepper lines used in this study. Top two lines (red cultivars): (A) CK6, (B) CK8, (C) R29, (D) R37, (E) R28, (F) R30, (G) R26, (H) R38; bottom two lines (yellow cultivars): (I) P123-1-1, (J) CK4, (K) CK4-1, (L) CK18, (M) CK7, (N) CK26-1, (O) R12, (P) R19.

All 156 pepper cultivars were grown in a field to examine the mature fruit colors. Most of the cultivars showed a diverse degree of red coloring in fruit, which included dark red, red and light red (Figure 1). In addition, some lines producing orange or brown ripe fruit, and eight cultivars producing yellow ripe fruit, were also observed (Figure 1). Since six lines with yellow ripe fruit were local peppers, cluster analysis was necessary to identify the genetic relationship between these lines and to determine which *Capsicum* species these lines belong to.

**Figure 2:**
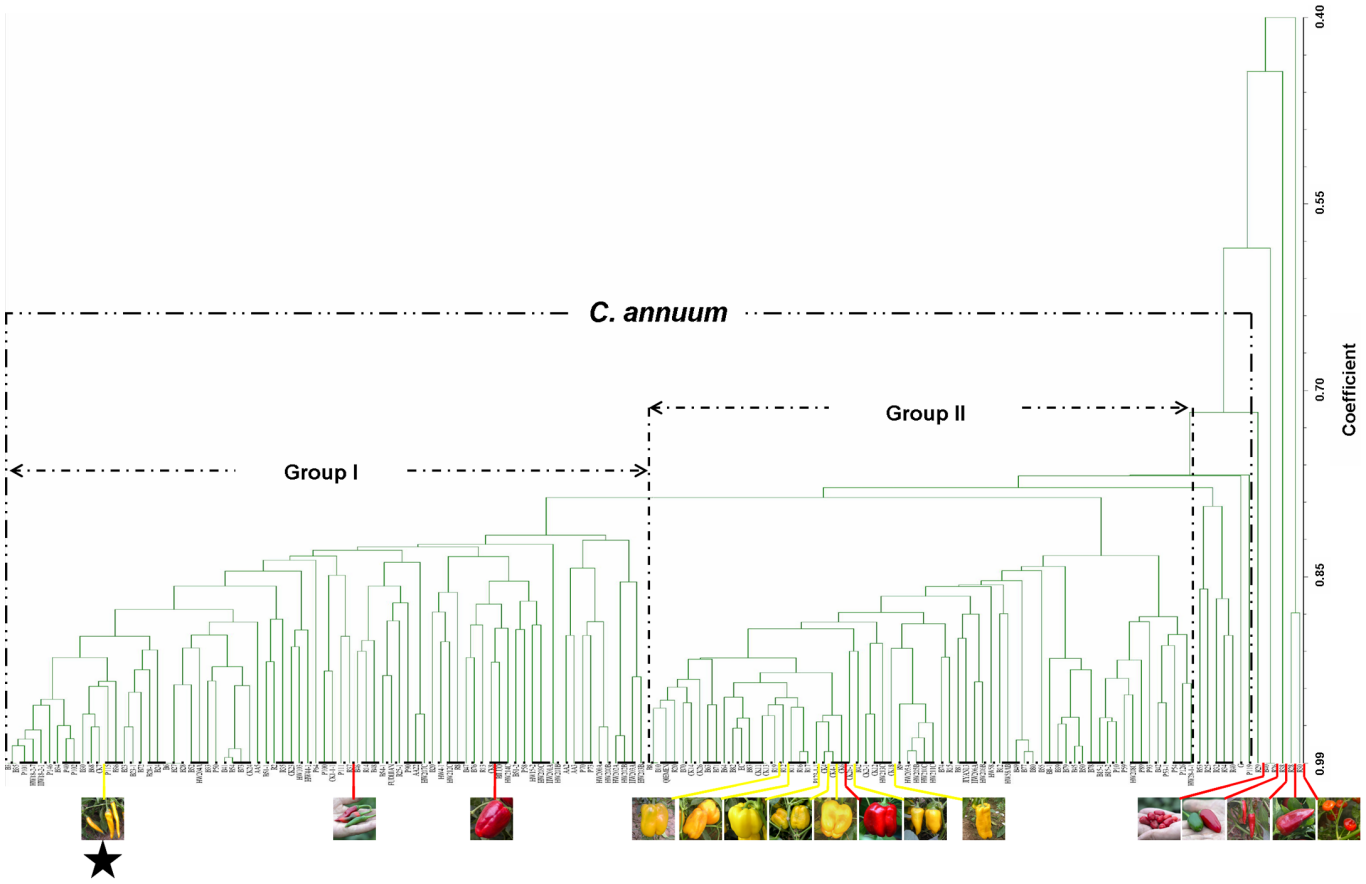
The clustering pattern obtained for all 156 pepper cultivars examined in this study using SSR analysis. The novel yellow ripe-color pepper line, CK7, is marked with an asterisk.

Across all the 156 cultivars evaluated, 32 SSR markers were analyzed, and 154 scorable polymorphic bands were detected. An UPGMA dendrogram (Figure 2) revealed one main cluster (*C. annuum*) and four genetically distinct species. The majority of the cultivars evaluated fell into the main cluster, which could be subdivided into two large (I, II) and one small group. All of the unknown local yellow pepper lines were grouped in the main cluster, revealing that these lines are all *C. annuum* cultivars. Among the eight yellow cultivars, seven are in group II, which mainly includes sweet or bell pepper. One exception is the yellow fruit line CK7, which is found in group I. Most chili peppers in this study are found in this group, and CK7 is also a hot pepper. Therefore, the genetically distinct background between CK7 and other yellow cultivars suggests that the yellow color fruit genotype may have an independent mutational origin.

### Sequence diversity in the coding sequence of the *Ccs* allele

**Figure 3:**
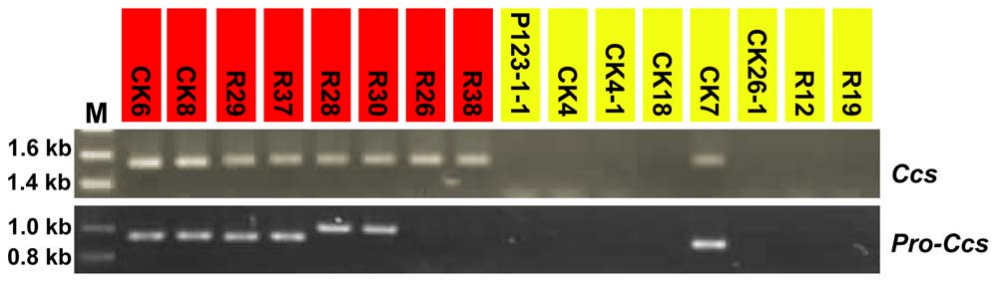
PCR amplification of the *Ccs* gene and its promoters from the genomes of the indicated *Capsicum* varieties. The order of the pepper lines is the same as in Figure 1, and the mature fruit colors are indicated with red and yellow highlighting.

**Figure 4:**
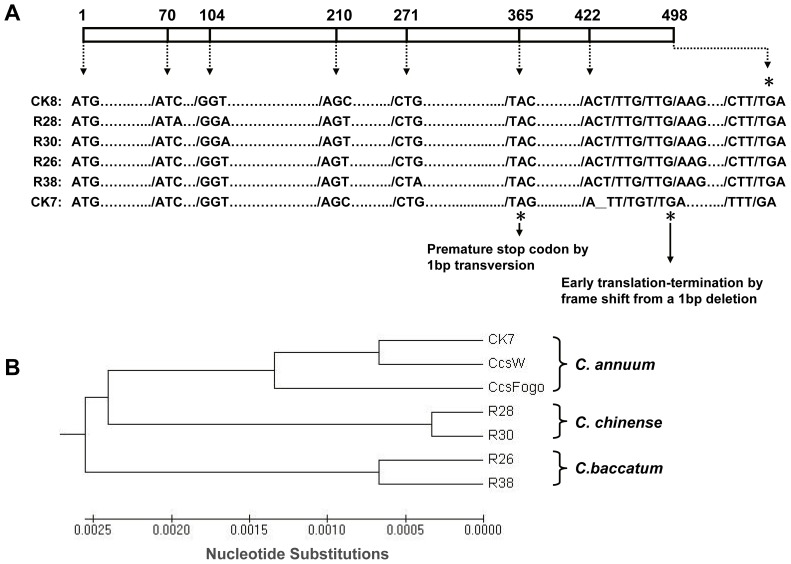
Comparisons of the *Ccs* coding sequences in the indicated *Capsicum* varieties. (A) Schematic representation of the mutations in *Ccs* among different cultivars. The nucleotide sequences were aligned, and the resulting missense mutations in the amino acid sequence are marked with asterisks. The 1-bp deletion in the coding sequence, which leads to early translation-termination, is underlined in the CK7 sequence. ATG and TGA indicate the start and stop codons, respectively. (B) Phylogenetic tree of the *Ccs* gene generated by multiple alignments of the coding sequence. The sequence of CcsW, which served as the positive control, was obtained from the NCBI (Accession: ×76165), and the sequence of CcsFogo was obtained from Guzman *et al*. [11] (GenBank GU122933).

PCR was performed to analyze the genomic coding sequences of *Ccs* in the 156 pepper varieties. All non-yellow lines produced positive PCR results after electrophoresis (Figure 3 showed a part of the results). As expected, we did not detect the *Ccs* gene in seven yellow color cultivars, which is agreement with the results of Lefebvre *et al*. [13]. However, one yellow line, CK7, produced the same banding pattern as that of the red cultivars (Figure 3). The PCR band from CK7 was extracted from the gel and sequenced. Sequence analysis revealed that CK7 contains a new variant of *Ccs*, with two types of early translation termination of the *Ccs* gene (Figure 4A). One mutation is a frame shift derived from a 1-bp deletion (1,265 bp downstream of the start codon), which is the same as the mutation detected in the *C. annuum* cultivar Fogo [11]. The second mutation, a novel premature stop codon produced by a point mutation (1,095 bp downstream of the start codon, cytosine to guanine) was found upstream of the deletion described above.

The premature stop codon in the *ccs* gene in CK7 encodes a putative 364 amino-acid truncated protein that lacks the 134 amino-acid region found in the C-terminus of the wild-type protein. The estimate molecular weight and isoelectric point (pI) of the mutant CCS protein are 41.4 kDa and 8.47, respectively, in contrast to 56.6 kDa and 8.77, respectively, in the wild type. *Ccs* belongs to the *lycopene cyclase* gene family (LYC), which encodes lycopene beta and epsilon cyclase proteins. Alignment of putative proteins encoded by several genes from this family, which share high homology with CCS, revealed that the amino acids at the C-termini are more conservative than those at the N-termini (Figure S1). The last deduced domain, which is located near amino acids 430–460, is totally lacking in the *ccs* variant of CK7.

The *Ccs* CDS regions from five other cultivars were also sequenced to analyze the structural polymorphism in this gene (Figure 4A). Among the cultivars, CK8, which belongs to *C. annuum* species and exhibits typical wild red fruit phenotype, was used as a positive check; four other lines (R28, R30, R26 and R38) were chosen due to their different promoter profiles (as described below). Some polymorphic nucleotides were found, whereas no changes in the amino acid sequence were detected in the four lines examined (Figure 4A). Together with the *ccs* sequence from CK7 and Fogo [11], a phylogenetic tree examining the *Ccs* gene was generated by multiple alignments of the nucleotide sequences in the CDS region (Figure 4B). Despite the presence of several mutations, the gene sequences from CK7, Fogo and wild *C. annuum* were still grouped into a same cluster, in contrast to the *C. chinense* and *C. baccatum* species. This suggests that an independent evolution and mutation may have occurred in the *Ccs* gene after *Capsicum* interspecies differentiation.

### Cultivar-specific *Ccs* expression

**Figure 5:**
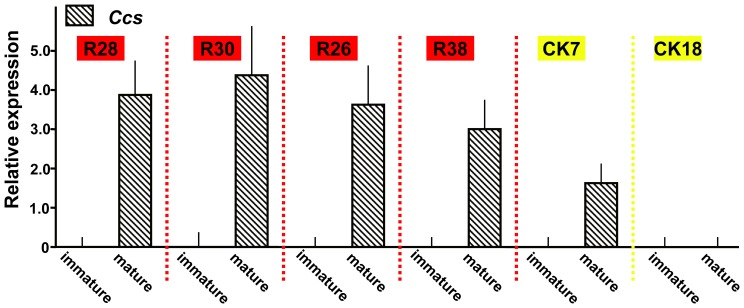
Expression pattern of *Ccs*. Total RNA obtained from immature and mature pericarps of R28, R30, R26, R38, CK7 and CK18 was used for quantitative RT-PCR analysis. Each result represents the average of three independent biological replicates ± SE (n = 3), with a significance level of P≤0.05.

Given the variability in gene structures and mature color phenotypes, we used quantitative RT-PCR to test the expression of *Ccs* in the lines that produced positive PCR results for the *Ccs* CDS (Figure 5). Transcripts for *Ccs* were not detectable in the immature fruits of any of the pepper lines examined (Figure 5). In the four red fruit lines, including two *C. chinense* (R28 and R30) and two *C. baccatum* lines (R26 and R38), *Ccs* expression was detected in fully mature fruits. We also detected some accumulation of *Ccs* transcript in CK7, which has a mutant *ccs* gene and yellow fruit, although the expression level was lower than that detected in the red fruit lines; a similar pattern was revealed in the Fogo accession [12]. As expected, none of the other yellow lines exhibited *Ccs* transcript accumulation.

### Structural variations in the *Ccs* promoters of different species

As the expression patterns for *Ccs* gene were variable across the pepper cultivars [4], [12], we examined the promoter regions of this gene in all of the varieties using PCR (Figure 3). All red mature *C. annuum* lines produced a similar 920-bp band as the result submitted in GenBank (Y14165). Two larger bands were detected in two *C. chinense* species (R28 and R30), and no PCR product was detected in two *C. baccatum* accessions (R26 and R38). The yellow mutant cultivar evaluated in this study, CK7, produced the similar PCR band as in red-fruited *C. annuum* lines, while none of the other yellow peppers produced a positive PCR result. The promoters from CK8 (used as a positive check for red ripe-fruit lines in *C. annuum*), CK7, R28 and R30 were sequenced, and no sequence differences in the two *C. annuum* lines (CK8 and CK7) were likely to be associated with the differences in *Ccs* gene transcription.

**Figure 6:**
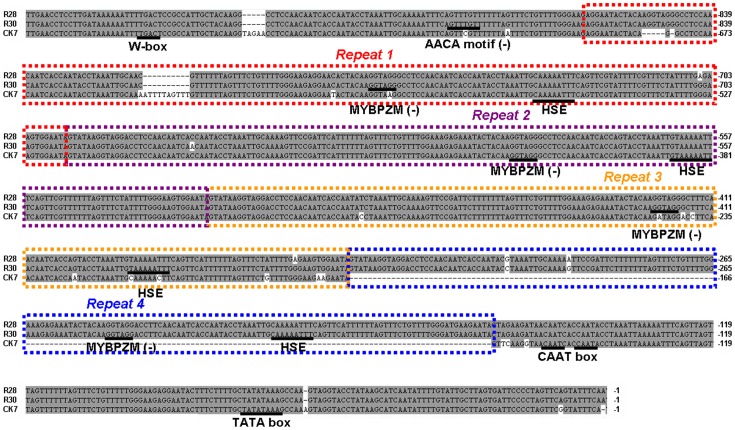
Sequence comparisons in the *Ccs* promoter region among the *Capsicum* varieties examined in this study. The boxes (red, purple, yellow and blue) indicate the four repeat units found in this region. Major *cis*–elements as predicted by PLACE and PlantCARE softeare are underlined.

After multiple alignments of the nucleotide sequences, some similar insertions/deletions in the promoter sections from different species reported by Ha *et al*. [4] were confirmed, including a 11/10-bp deletion and 176-bp insertion in the *C. chinense* lines (Figure 6). Unexpectedly, we found a tandem repeat structure in the promoter region, with the 176-bp insertion/deletion comprising just one unit of the repeat. The start position of this repeat structure is 165-bp upstream of the transcriptional start point. In addition, three continuous repeats (Repeat 1–Repeat 3; Figure 6) were revealed in the *C. annuum* lines, whereas four units (Repeat 1–Repeat 4) were detected in the *C. chinense* accessions. In the *C. chinense* accession R28, the similarity of the four repeat units ranged from 84.8% (Repeat 1 and Repeat 4) to 97.7% (Repeat 3 and Repeat 4) (Figure S2).

Various potential *cis*-regulatory elements were identified; the most notable ones are listed in Table 2 and underlined in Figure 6. Analysis of the 176 bp repeat unit sequence indicated that this sequence contains a heat stress-related *cis*-element (HSE, A/TAAAAATTTC) and an *Myb* homolog binding site (MYBPZM, CCT/AACC). In maize, the *Myb* binding site is a target recognized by the product of the maize *P* gene, which is associated with red pigmentation of the kernel pericarp and flavonoid biosynthesis [21].

**Table 2:** Specific putative *cis*-acting elements in the promoter of *Ccs* from *C. chinense* accession.

		Position					
Response/Binding Site	Sequence (5′ to 3′)	Region I^a^ (−1∼−165)	Repeat 4^b^ (−166∼−341)	Repeat 3 (−342∼−517)	Repeat 2 (−518∼−693)	Repeat 1 (−694∼−869)	Region II (−870∼−977)
Heat stress response	CCAATBOX1[CCAAT]		−223, −311	−399, −487	−663	−751, −838	−909
Heat stress response	YAAAAATTTC		−204	−380	−556	−732	
Light response	IBOXCORE[GATAA]						−961
Light response	INRNTPSADB[YTCANTYY]		−193, −198	−369, −374	−550, −633	−726	
Light response	SORLIP2AT[GGGCC]				−591		
Low-temperature response	LTRE1HVBLT49[CCGAAA]		−265^c^				
MYB binding site	MYBZM[CCWACC]		−330 c	−506 c	−682 c		
SEF4 binding site	SEF4MOTIFGM7S[RTTTTTR]	−129 c	−206 c	−382 c	−558 c	−734 c	
SPBF binding site	SP8BFIBSP8BIB[TACTATT]		−339 c	−515 c	−691 c		
TATA box	TATATAA	−70					
CAAT box	CAAT	−144, −151					
W box	TTTGACY						−949

Note: negative values indicate the first nucleotide position of the *cis*-element with respect to the transcription initiation site located at position +1.

^*a*^The promoter of *Ccs* from *C. chinense* accession is divided into six parts, which consists of four tandem repeat (Repeat 1 - 4) flanked with proximal (Region I) and 5′-distal (Region II) sequence.

^*b*^Repeat 4 is lacking in *C. annuum* accession.

^*c*^Sequence of the complementary strand.

## Discussion

Since *Capsicum* exhibits variations in morphology and genetic composition, many studies of this plant involving cluster analysis with different molecular markers systems have been reported [22]–[26]. In these studies, AFLP analysis was useful for revealing high levels of polymorphism, while SSR method was useful for detecting specific genetic relationships between pepper cultivars [23], [25]. Here, we selected 32 SSR loci, including seven markers used by INRA (France), to perform cluster analysis of all of our local pepper cultivars. In this study, 32 SSR markers produced 154 polymorphic bands, with an average allelic variation of 4.8. In addition to *Capsicum* species, we could also distinguish the major chili and bell pepper lines among *C. annuum* cultivars. In fact, we failed to detect genetic difference between only five pairs of pepper lines (Figure 2). Among these pairs, B3 and B35, B6 and B27, B8 and B10, CK11 and CK13 were all selected from a same locality in Northwest China, with similar morphologies, which suggests that both lines in each pair are closely related. The remaining pair, HW201A and HW201B, is a male sterile line and its maintainer. This study revealed that, in addition to the AFLP method, SSR analysis is a powerful method for analyzing the phylogenetic relationship between *C. annuum* species.

The complex taxonomic relationship among *Capsicum* species implies that diverse genetic variation can lead to similar phenotype. To date, the heredity of mature pepper fruit color is still not fully understood. In the orange-fruited cultivars, ripe fruit color involves not only one or two structural changes in the genes of the carotenoid biosynthetic pathway, but also the transcriptional and/or post-transcriptional regulation of these genes [12]. However, in the yellow ripe-color pepper lines, fruit color is determined by variations in the *Ccs* gene [4], [7], [13]. Three structural mutations in the *Ccs* gene have thus far been identified in yellow fruit pepper lines. The first mutation involves a deletion in the gene region, which maybe didn’t contain the distal 220 bp of the 3′ end of this gene in yellow *C. annuum* cultivars [7], [13]. The second and third mutations, which involve a premature stop codon and a frame-shift, respectively, were detected in two *C. chinense* accessions [4]. Similar mutations in the *Ccs* gene in *C. annuum* and *C. chinense* have not previously been reported. Here, using cluster analysis, we determined that a local yellow-colored ripe fruit cultivar, CK7, is a *C. annuum* line that harbors a new *ccs* variant. In CK7, the gene has the same frame-shift mutation in CDS as identified in Fogo, an orange-colored mature *C. annuum* accession [11]. The mutant *ccs* variant in Fogo could result into a truncated production, and prevented the synthesis of capsanthin and capsorubin [12]. In addition, the *ccs* gene shares a similar expression pattern in both lines, which differs from the expression pattern detected in the yellow-colored mature *C. chinense* accessions [4]. These results suggest that there is a close genetic relationship between Fogo and CK7. Considering the different fruit colors exhibited in these two lines, the mutation in the *Ccs* gene may have occurred before the modified regulation of the gene, at least in the Fogo and CK7 accessions. Furthermore, the mutation that produced the yellow fruit phenotype may have occurred prior to the post-regulation detected in orange-colored mature fruit pepper. Additionally, a special upstream premature codon was found in CK7, whose appearance can be explained by the theory of high rate random mutation in a nonfunctioning gene.

Since the regulation of gene expression is another important area of investigation in the analysis of pepper fruit color, we thought it was important to examine the promoters of the *Ccs* gene. Ha *et al*. [4] identified three classes of promoter sequences in three *Capsicum* species and suggested that differences in this region reflect species-specific variations in *Capsicum*. We repeated their experiment, and PCR amplifications in two *C. baccatum* accessions (R26 and R38) yielded negative results (Figure 3). This may have been due to the different pepper varieties examined between both studies, as well as the complex genetic background of pepper. We detected the longer amplicons in the *C. chinense* lines (R28 and R30) that were also found by Ha *et al*. [4]. Sequence analysis revealed a unique tandem repeat structure in the *Ccs* promoter region. The tandem repeats comprise a major part of the promoter (65%, 528/812 bp in the amplicon from *C. annuum*) and are located near the transcriptional start point (165-bp upstream). Therefore, the presence of putative *cis*-elements, and the evolutionary significance of the repeats, cannot be ignored. Using bioinformatics, we detected some light and temperature sensitive elements in every repeat unit (Table 2), which may explain why the accumulation of capsanthin/capsorubin increases in response to abundant sunshine and high temperatures.

The proximal 176-bp tandem repeat structure in promoters is very rare in plants. The presence of this structure may have led to the deletion of the *Ccs* gene in most yellow-colored fruit *C. annuum*. Since the *Ccs* promoter is functional in tomato [27], it would be both valuable and feasible to study the effects of the proximal 165-bp promoter region and repeat unit on the transcriptional regulation of *Ccs*.

## Supporting Information

Figure S1 **Alignment of the deduced amino acid sequence of CCS and its homologous proteins.**
(DOC)

Figure S2 **Sequence comparisons of the four repeat units in the** ***Ccs*** **promoter region.**
(DOC)

Table S1 **Simple sequence repeat (SSR) loci used in this study.**
(DOC)

Table S2 **Gene-specific primers used in this study.**
(DOC)
