# Supplementary material for: A Further Analysis of the Relationship between Yellow Ripe-Fruit Color and the Capsanthin-Capsorubin Synthase Gene in Pepper (Capsicum sp.) Indicated a New Mutant Variant in C. annuum and a Tandem Repeat Structure in Promoter Region
Source: PLoS One. 2013 Apr 18;8(4):e61996. doi: 10.1371/journal.pone.0061996 (PMC3630222; doi:10.1371/journal.pone.0061996)
Supplement: Figure S1 — Alignment of the deduced amino acid sequence of CCS and its homologous proteins. (DOC) [file pone.0061996.s001.doc]

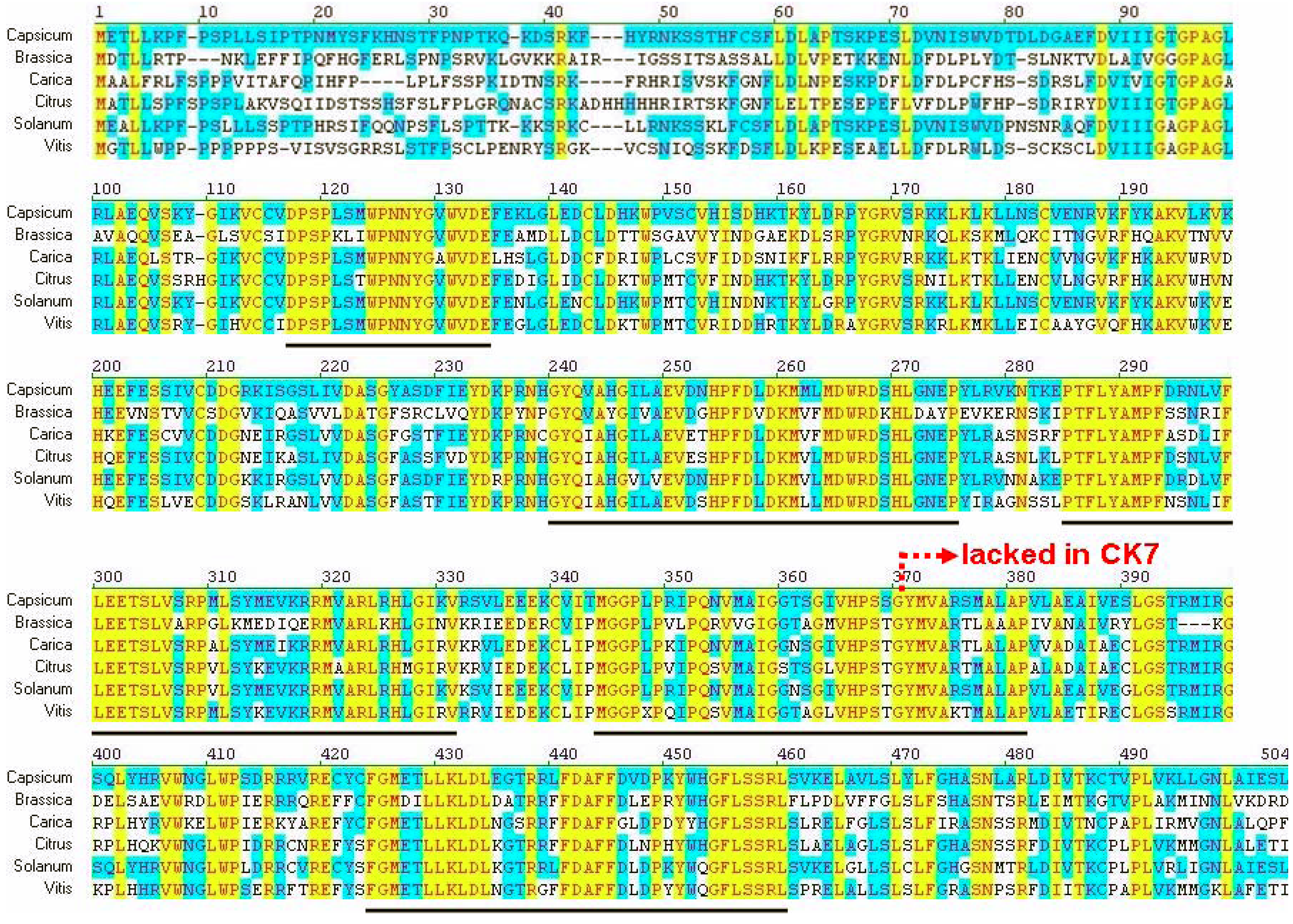


Figure S1. Alignment of the deduced amino acid sequence of CCS and its homologous proteins. The genes are *Brassica napus* (ADT89710, chloroplast lycopene beta-cyclase), *Carica papaya* (ACR61334, lycopene beta-cyclase 2), *Citrus x paradisi* (ACR09635, lycopene beta-cyclase), *Solanum lycopersicum* (NP_001234445, chromoplast-specific lycopene beta-cyclase) and *Vitis vinifera* (AFP28803, lycopene beta-cyclase 1). The region lacked in the truncated production derived from CK7 in this work was designated by an arrow. Some predicted conserved domains were *underlined*.
