# Supplementary material for: A Further Analysis of the Relationship between Yellow Ripe-Fruit Color and the Capsanthin-Capsorubin Synthase Gene in Pepper (Capsicum sp.) Indicated a New Mutant Variant in C. annuum and a Tandem Repeat Structure in Promoter Region
Source: PLoS One. 2013 Apr 18;8(4):e61996. doi: 10.1371/journal.pone.0061996 (PMC3630222; doi:10.1371/journal.pone.0061996)
Supplement: Figure S2 — Sequence comparisons of the four repeat units in the Ccs promoter region. (DOC) [file pone.0061996.s002.doc]

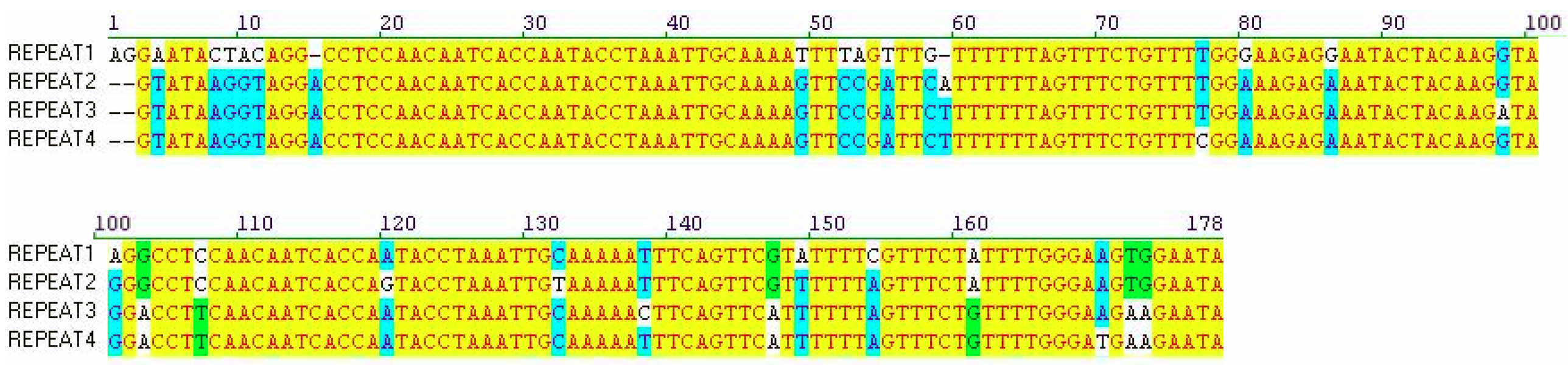


Figure S2. Sequence comparisons of the four repeat units in the *Ccs* promoter region. Repeat 1~3 can be found in all *Capsicum* varieties analyzed here, and repeat 4 are unique in *C. chinense* accessions in this study.
