# Supplementary material for: A Further Analysis of the Relationship between Yellow Ripe-Fruit Color and the Capsanthin-Capsorubin Synthase Gene in Pepper (Capsicum sp.) Indicated a New Mutant Variant in C. annuum and a Tandem Repeat Structure in Promoter Region
Source: PLoS One. 2013 Apr 18;8(4):e61996. doi: 10.1371/journal.pone.0061996 (PMC3630222; doi:10.1371/journal.pone.0061996)
Supplement: Table S1 — Simple sequence repeat (SSR) loci used in this study. (DOC) [file pone.0061996.s003.doc]

Table S1. Simple sequence repeat (SSR) loci used in this study

| SSR locus | Forward primer (5’ to 3’) | Reverse primer (5’ to 3’) | Linkage group |
| --- | --- | --- | --- |
| CAMS020 | CAGCAGTAACAGAGGCAGGTC | CACAAGTGAGTTTATTCATATCACCA | 5 |
| CAMS024 | TGTTGAGGCTTGGGAAAAAC | TGTTGAGGCTTGGGAAAAAC | 8 |
| CAMS051 | ACCCAGTTCCCTTTCTTGGT | GAAGGTTAGCGGAATGAACG | 5 |
| CAMS065 | CCAGTCTCATCCAGCAGACA | CATATGCTGCTCCTGCATTC | 2 |
| CAMS075 | ACTAATTACACATTCTGCATTTTCTC | AGGCTCGAGTACCACGAAGA | 5 |
| CAMS090 | TCGCTCAAAGCACATCAAAG | CTTGATTGTTCTTCCACTGCTG | 8 |
| CAMS095 | CGCTAGCATGACACTCAAGG | AAACGGCAAGGCTACACATC | 5 |
| CAMS142 | GAGCGCTTAAGTGGTCATAGG | CTACAACGCCCCAAAACAAT | 7 |
| CAMS156 | CCCTATGCTTTCACAACTCCT | GACGTGGTTATGACGATAGGC | 10 |
| CAMS162 | GGACCGTTCAGGAGGTTACA | GCCATCATTCAAAACCGAAT | 1 |
| CAMS163 | TCCATATAGCCCGTGTGTGA | GCGTGGGAATACAATGCTAGA | 5 |
| CAMS177 | ATTCTCTACCCCTGCCTGTG | CTCAGGAGATGTCCCACGAT | 2 |
| CAMS227 | TTTGTCCTTTAATTCACCTTTTGA | GCATCAAAATAAGGATAAAGTTATGG | 5 |
| CAMS313 | CAGCCTGCTTGGCTAGAACT | TCGTCATGCATGGCTAATCT | 11 |
| CAMS321 | CTGCAGGTGGTTCTCTCCAT | TGGCACTCGAACCAGTATGA | 3 |
| CAMS327 | GCATCTAAGTCTACGCCCTTG | AAAGCCTTTGGCAATGAACA | 13 |
| CAMS348 | CTGAAGTCGGCTAGATGCCTA | TCAAAGCTATGGAGGAAAAGGA | 3 |
| CAMS361 | TTGGTGTGGTTAGGGGAGAG | GGCGTTCGAACTTGTGAAAT | 4 |
| CAMS378 | GAAATCGACGCGTTTCTAGC | TGTGGGGAGAGAGAGGAAGA | 1 |
| CAMS405 | TTCTTGGGTCCCACACTTTC | AGGTTGAAAGGAGGGCAATA | 11 |
| CAMS417 | CCCAAAGACGATTTGCTGA | ATTGCCTGTGAGTGCAACAA | 10 |
| CAMS420 | CAGCGTTCTATCGTCTCAAATG | TTGACAAACCAGAAATTGATCG | 5 |
| CAMS424 | TCCACAGCCCACAGTGTCTA | GCTTGTGGTTCCGTGATTTT | 6 |

(Table S1 continued)

| CAMS451 | TGCATTGGTGGGCTAACATA | GCTCTTGACACAACCCCAAT | 11 |
| --- | --- | --- | --- |
| CAMS606 | GACTAGTCCCCGTTCAACCA | TTTGCGAGAAGATGCTTCAG | 7 |
| EPMS310* | TGGGAAGAGAAATTGTGAAAGC | AGGAAACATGGTTCAATGCC | 8 |
| EPMS342* | CTGGTAGTTGCAAGAGTAGATCG | ATGATCTTTGACGACGAGGG | 8 |
| EPMS391* | TTTCTTCTCTGGCCCTTTTG | ACGCCTATTGCGAATTTCAG | 11 |
| GPMS197* | GCAGAGAAAATAAAATTCTCGG | CAATGGAAATTTCATCGACG | 12 |
| GPMS29* | CAGGCAATACGGAGCATC | TGTGTTGCTTCTTGGACGAC | 11 |
| GPMS6* | CAGAGCACTTGACATGCCTT | GATCTTTATAGTAGCTCATCAATA | 2 |
| HPMSE013* | GCGCCAAGTGAGTTGAATTGAT | CACCAATCCGCTTGCTGTTGTA | 10 |

Note: the locus marked with * were provided from Prof. Alain Palloix (INRA, France); other SSR markers were according with Minamiyama *et al*. [15].
