# Supplementary material for: A Further Analysis of the Relationship between Yellow Ripe-Fruit Color and the Capsanthin-Capsorubin Synthase Gene in Pepper (Capsicum sp.) Indicated a New Mutant Variant in C. annuum and a Tandem Repeat Structure in Promoter Region
Source: PLoS One. 2013 Apr 18;8(4):e61996. doi: 10.1371/journal.pone.0061996 (PMC3630222; doi:10.1371/journal.pone.0061996)
Supplement: Table S2 — Gene-specific primers used in this study. (DOC) [file pone.0061996.s004.doc]

Table S2. Gene-specific primers used in this study

| Analysis | Forward primer (5*’* to 3*’*) | Reverse primer (5*’* to 3*’*) | Reference |
| --- | --- | --- | --- |
| Cloning of *Ccs* CDS | CCTTTTCCATCTCCTTTACTTTCCATT | AAGGCTCTCTATTGCTAGATTGCCCAG | [13] |
| Cloning of *Ccs* promoter fragment | TTGAACCTCCTTGATAAAA | GGAAAGTAAAGGAGATGGA | [4] |
| RT-PCR for *Ccs* | ATAGAGTTTGGAATGGTTTGTGG | CCTAGCCAAATTAGAGGCATGT | [4] |
| RT-PCR for *ubiquitin* in pepper | AATAAGGATGCAGGCTTCAAGGGC | TGATGTCACGGGACCGAAGAAGAT | [20] |
